# Dislocation of the Spleen

**Published:** 1873-09

**Authors:** A. R. Kilpatrick

**Affiliations:** Navasota, Texas


### DISLOCATION OF THE SPLEEN.

BY A. R. KILPATRICK, M.D., OF NAVASOTA, TEXAS.

The displacement, or dislocation of the spleen occurs so rarely, and is so seldom mentioned by authors, that probably some of the readers of the RECORD may have their doubts of there being such a pathological fact.

Dr. Robley Dunglison, in his "Practice of Medicine," mentions one case he saw *post mortem*, and reports it as a rarity. He found the spleen broken loose from its attachments, resting with its convex surface on the brim of the pelvis—the lower extremity being turned up in the right lumbar region, suspended by its vascular and peritoneal attachments, and easily movable in every direction.

In April, 1867, I attended a young widow here who was sick with intermittent fever, complicated with deranged uterine function. She was the mother of two children, and about twenty-five years old. She had lived in Eastern Texas, and suffered for years with different diseases, mostly, though, malarial fevers. She was poor, and had to cook and do all household work. In course of treating her case, I examined the abdominal contents, and as she was lean, the abdominal walls thin, all the viscera could be felt. I expected, on the first examination, to find a large spleen, but being disappointed, I asked if she had ever had "fever cake," and she said she had, years ago, and the doctors had treated her for it; but of late she had no trouble with it.

Pressing in the region of the uterus and bladder, I found the spleen there laying very much in the position given above by Prof. D. I am sure I was not mistaken: I found no sign of the organ in its proper place. The spleen could be easily traced out by the fingers, and was not much above the ordinary healthy size and length in the adult. Moving it or pressing on it produced no undue pain. She could give me no satisfactory accouut as to its dislocation. The woman moved away and died, I heard, of yellow fever, in the summer or fall, as there was an epidemic that year.

In June, 1868, while attending a black woman, who had several children, some of them grown, I found her spleen dislocated and laying exactly in the position of the foregoing case. She was also lean and thin, and the digital examination could easily trace out its size and situation. She also suffered no pain from handling, moving or pressing on the organ: it was evidently not diseased then, but she had no knowledge of when the dislocation occurred. She had been a slave, belonging to a sugar planter in Louisiana.

In 1866, a young lady of this place, who had a large spleen, was attempting to get up in a light wagon, when she was suddenly seized with a very sharp, lancinating pain in the spleen. She had to be helped back into the house, and required medical assistance. Dr. Barnett was called in, and found the spleen entirely below the ribs, leaving a space of more than two inches between the upper edge of the spleen and the ribs. It was very large, and occupied the pelvis, and felt like the gravid uterus. Her clothes were loosend, and her body placed on an inclined bed, elevating the posts so as to make the head much the lowest; then, by pressure and continued manipulation, similar to the taxis in reducing hernia, it was restored to its position and secured by bandages. She was kept in bed and the bandages attended to a few days, when she recovered entirely, and has not been troubled with the spleen since.

I have heard of another case in a married woman in Falls county, who is the mother of three children. She has suffered with enlarged and painful spleen several years. It finally has become dislocated, and now occupies the pelvis and feels like the gravid uterus. Since the location she has aborted several times—in fact, has not brought a child to term since. The spleen must weigh nine pounds, and, by its pressure on the uterus and on the large blood-vessels, has entirely impaired her health. The displacement came on gradually, and probably was facilitated by parturition.

These cases are reported partly as curiosities of medical experience, and partly to direct the attention of the profession so as to elicit further observation.

---

Tobacco Enema.—Dr. N. S. Davis, of Chicago, recommends for extreme cases of obstructions of the bowels an infusion of one drachm of chewing tobacco in a pint of boiling water, one-half or more to be injected when cool enough.

